# Molecular impact of launch related dynamic vibrations and static hypergravity in planarians

**DOI:** 10.1038/s41526-020-00115-7

**Published:** 2020-09-08

**Authors:** Nídia de Sousa, Marcello Caporicci, Jeroen Vandersteen, Jose Ignacio Rojo-Laguna, Emili Saló, Teresa Adell, Gennaro Auletta, Jack J.W.A. van Loon

**Affiliations:** 1 Department of Genetics, Microbiology and Statistics, Institute of Biomedicine, University of Barcelona (IBUB), Barcelona, Spain; 2 Life Support and Physical Sciences Section (TEC-MMG), European Space Agency—European Space Research and Technology Centre (ESA-ESTEC), Noordwijk, The Netherlands; 3 TEC-ECC, European Space Agency—European Space Research and Technology Centre (ESA-ESTEC), Noordwijk, The Netherlands; 4 Pontifical Gregorian University, Roma, Italy; 5 University of Cassino, Cassino, Frosinone Italy; 6 DESC (Dutch Experiment Support Center), Amsterdam University Medical Center location VUmc and Academic Centre for Dentistry Amsterdam (ACTA), Vrije Universiteit Amsterdam, Department of Oral and Maxillofacial Surgery/Pathology, Amsterdam Movement Sciences, Amsterdam, The Netherlands

**Keywords:** Biophysics, Developmental biology

## Abstract

Although many examples of simulated and real microgravity demonstrating their profound effect on biological systems are described in literature, few reports deal with hypergravity and vibration effects, the levels of which are severely increased during the launch preceding the desired microgravity period. Here, we used planarians, flatworms that can regenerate any body part in a few days. Planarians are an ideal model to study the impact of launch-related hypergravity and vibration during a regenerative process in a “whole animal” context. Therefore, planarians were subjected to 8.5 minutes of 4 g hypergravity (i.e. a human-rated launch level) in the Large Diameter Centrifuge (LDC) and/or to vibrations (20–2000 Hz, 11.3 *G*_rms_) simulating the conditions of a standard rocket launch. The transcriptional levels of genes *(erg-1*, *runt-1*, *fos, jnk*, and *yki*) related with the early stress response were quantified through qPCR. The results show that early response genes are severely deregulated after static and dynamic loads but more so after a combined exposure of dynamic (vibration) and static (hypergravity) loads, more closely simulating real launch exposure profiles. Importantly, at least four days after the exposure, the transcriptional levels of those genes are still deregulated. Our results highlight the deep impact that short exposures to hypergravity and vibration have in organisms, and thus the implications that space flight launch could have. These phenomena should be taken into account when planning for well-controlled microgravity studies.

## Introduction

There is currently an increased interest in space research and gravity-related sciences, especially since commercial space travels opened broad and new perspectives in medical and physical research, innovative tourism, or even in terms of territorial expansion towards the Moon and Mars. The 2005 NASA initiative to designate the US segment of the International Space Station (ISS) as a US national lab^1–3^ is in line with this trend. In this scenario, more research is required on the mechanisms of the effect of space flights on the function of cells and organisms. Fluctuations in gravity, vibration, pressure, temperature and radiation are the main parameters to take into account in space flight studies^4–6^. While many examples of how simulated and real microgravity, simulated and real partial gravity, and hypergravity demonstrate their profound effects on biological and physiological systems are found in literature, see for review^7–10^, a limited number of reports deal with specific launch-related hypergravity^11–13^ or vibration^13–16^ effects, the levels of which are severely increased in every space flight during ascent. There are, up till now, no ground-based research reports where the effects of launch vibrations and static g loads are applied simultaneously.

To learn of the effects of hypergravity and vibration in living organisms, we report on the impact of static and dynamic loads on planarians. Planarian are flatworms with the unique ability to regenerate any missing part of their body, even the head, after amputation^17,18^. Planarians show a centralized nervous system, with an anterior brain to which two nerve cords are connected, two eyes, a digestive system that connects to an evaginable pharynx, and an excretory system^18^. The amazing regenerative ability of planarians is due to the presence of a population of adult stem cells—called neoblasts—that are totipotent, and thus able to give rise to any planarian cell type^18–20^. Planarian plasticity is also visible during their normal homeostasis, since they continuously grow and degrow depending on food availability^18,21^. The presence of these unique adult stem cells and their plasticity renders planarians in a unique model to study the impact of environmental factors like hypergravity and vibration in adult cells in the context of a “whole animal”, in contrast to the partial view inherent to “in vitro” cell cultures. Also, there is an increasing interest to study these animals under various altered gravity conditions^22–26^.

Planarians were subjected to 4 g hypergravity in the Large Diameter Centrifuge (LDC) and/or to vibration for 8.5 minutes (20–2000 Hz, 11.3 *G*_rms_), simulating the conditions of a standard human-rated rocket launch. The transcriptional levels of genes related with the early response were quantified through qPCR. The results show that despite planarians regenerate apparently as good as controls, genes that respond just a few hours after wounding (early response genes) are significantly up- or down-regulated after the various treatments. Furthermore, the deregulation is higher after the combined exposure of static and dynamic g loads. Importantly, four days after exposure, the transcriptional levels of those genes are still deregulated. These results highlight the deep impact that launch-related hypergravity and vibrational loads can have on cells and whole organisms, and thus the implications for space flights experiment design and logistics.

## Results

### Planarians after short-duration vibration and/or 4 g hypergravity are able to properly regenerate the missing head

**Fig. 1:**
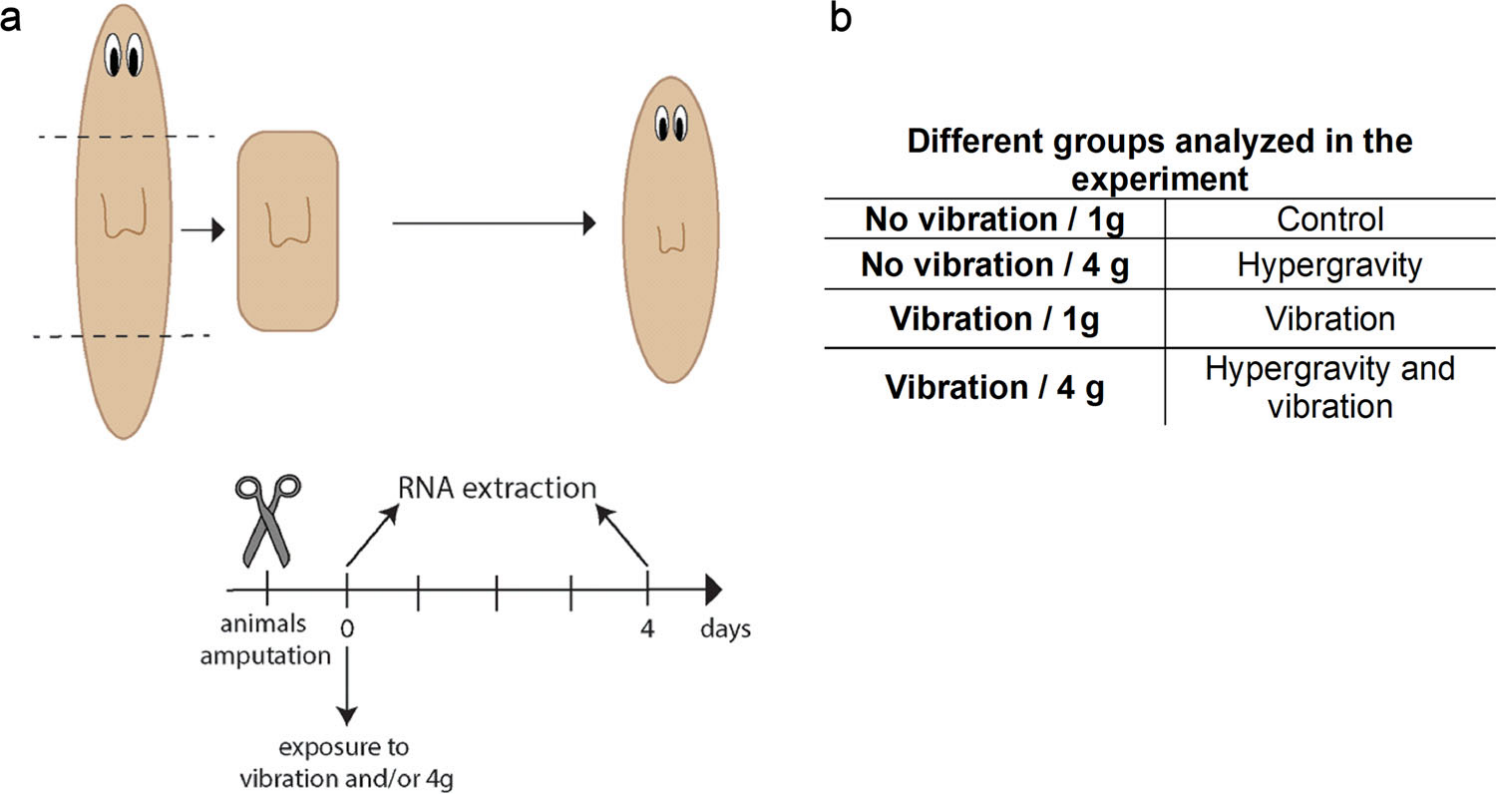
Experimental design. **a** Animals were amputated the day before of the exposure to simulated launch loads (day −1). At day 0, animals were loaded into T25 flasks and the experiment was initiated. Immediately after the exposure to hypergravity, vibration or both, RNA was extracted from half of the animals (0 h). Four days after the exposure, the RNA of the rest of the animals was extracted (4d). The regenerated structures were imaged at 4 days after the exposure. **b** The four experimental groups of animals.

**Fig. 2:**
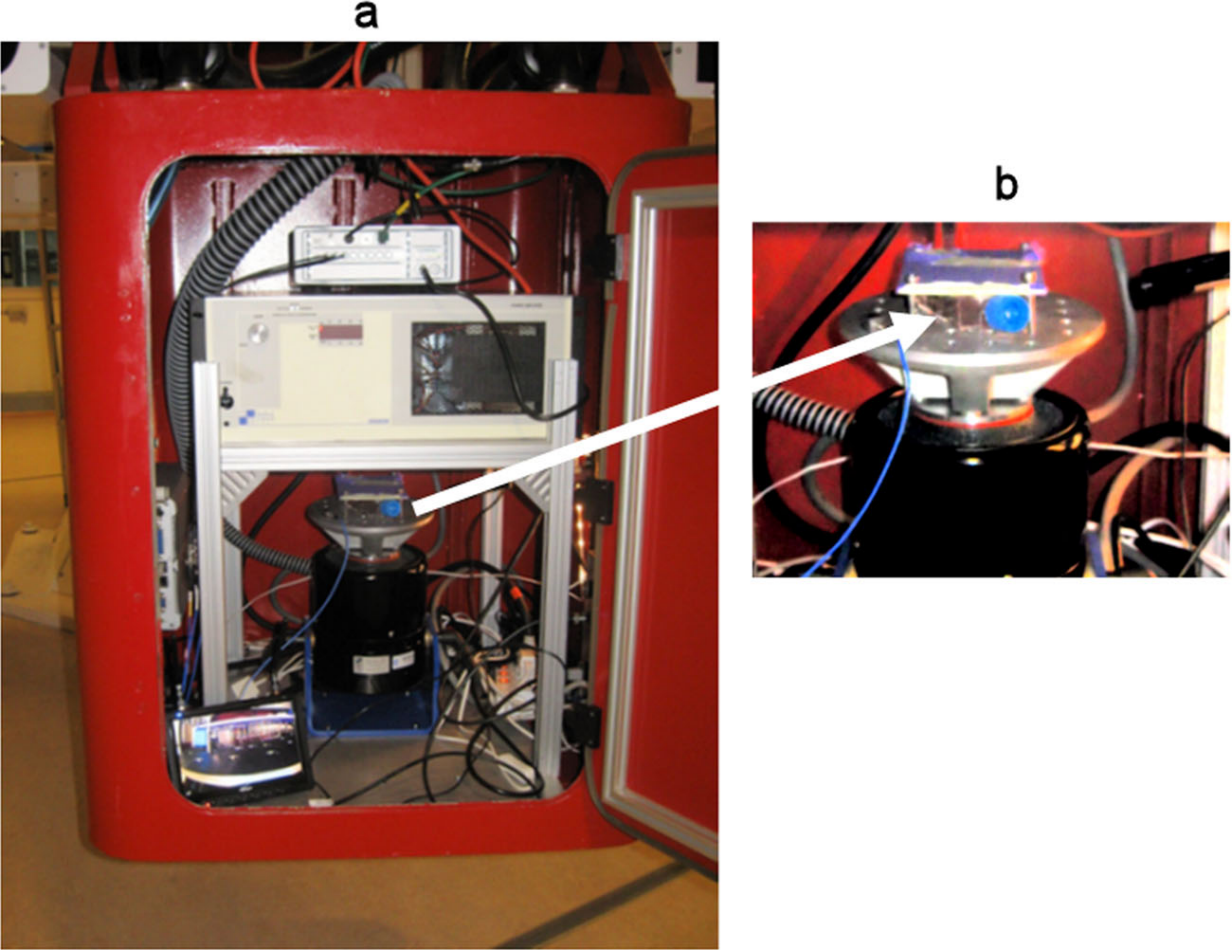
Vibration system. **a** Inside of the LDC gondola the vibration system consisting of the actuator, the amplifier and the data acquisition system was mounted in one gondola. The cooling system was placed in a second gondola (not visible in the image). **b** Detail of the top part of the actual actuator shown here with a T25 flask attached which contained five animals. The flasks were completely filled with planarian artificial medium, leaving no air bubbles. During simulated launch exposures the animals were at ambient conditions. During other periods the temperatures were 20–22 °C.

In order to verify whether vibration and hypergravity conditions affects planarian regeneration, 1-day-regenerating planarian trunks - the head and the tail were amputated the day before - were loaded into the LDC in which a vibration system was adapted (Figs. 1a and 2).

**Fig. 3:**
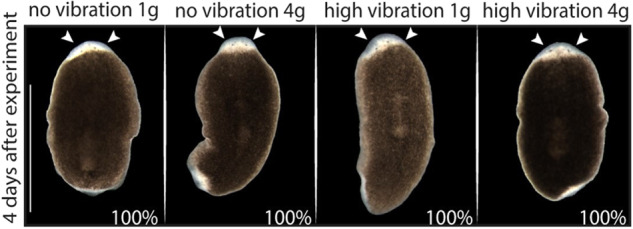
In vivo phenotype of animals exposed to hypergravity and/or vibration 4 days after the exposure. Animals in all groups were able to regenerate the head (the eyes are indicated with arrow heads). No alterations are observed between the animals from the four different conditions. *n* ≥ 10. Scale bar = 1 mm.

To explore the effects of hypergravity and vibration individually and combined, four animal groups were included: planarians at 1 g without vibration (control group), planarians at 4 g without vibration, planarians at 1 g with vibration, and planarians at 4 g with vibration (see Fig. 1b). In vivo observation of the animals immediately after the treatment (0 h) and 4 days (4d) after showed no obvious difference in the regenerative abilities between the various planarian groups (Fig. 3). Thus, planarians under vibration and/or 4 g hypergravity, regenerate an apparently proper head.

### Planarians after short-duration vibration and/or 4 g hypergravity show a transcriptomic deregulation of genes essential for regeneration

**Fig. 4:**
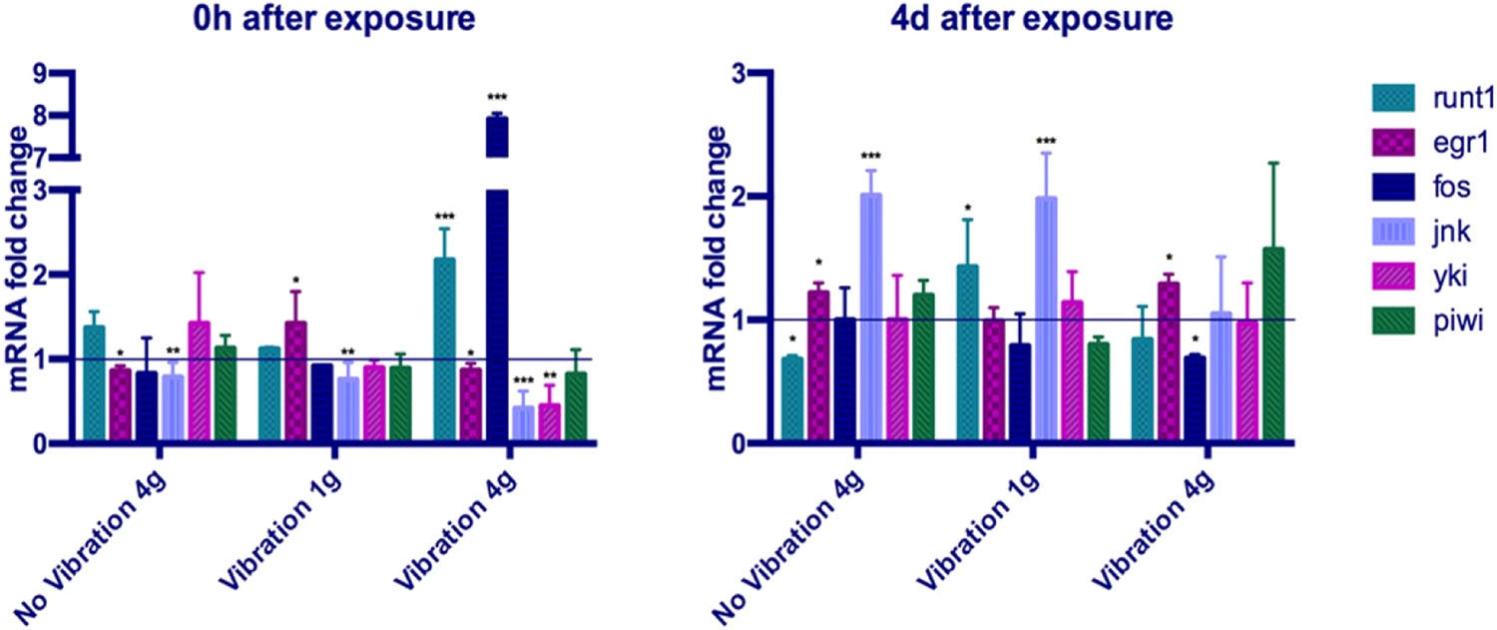
qPCR analysis of planarians exposed to vibration or/and 4 g hypergravity compared to 1 g static controls directly after exposure or at 4 days post exposure. The mRNA levels of the indicated genes are analyzed with respect to the levels of ura4. Values represent the means of at least two biological replicates each one with five animals. Error bars represent standard deviation. Data was analyzed by two-sided Student’s *t*-test. *p < 0.05; **p < 0.01; ***p < 0.001.

In planarians, the onset of regeneration relies on the transcriptional activation of early response genes, which are known to be quickly activated after any kind of wound. Those genes are essential for the proper proliferation and differentiation of stem cells after an injury^27,28^ in order to restore the missing structures. We performed a qPCR analysis to explore the expression levels of specific early response genes in the animals corresponding to the four conditions studied. We analyzed the expression of the transcription factors *runt-1*, which is normally expressed 3–6 h after wounding and is required for specifying different cell types during regeneration^27,28^ and *egr-1* (*early growth response-1*), which is expressed 1 h after wounding^27^ (Fig. 4). We also analyzed the expression of JNK pathway-related genes (*fos-1*, *jnk*), which coordinate the apoptotic and the mitotic response required for proper regeneration^29^ (Fig. 4). Our results show that immediately after the exposure (0 h) planarians that were subjected to only a static load of 4 g show a significant decrease in the transcriptional levels of *egr-1* and *jnk*. Planarians which were subjected to only a dynamic load of a random vibration showed a significant increase in the transcriptional levels of *egr-1* and a decrease of *jnk*. The most interesting result was that the simultaneous exposure of planarians to 4 g hypergravity and vibration severely affected the transcription of the early genes. Thus, five genes analyzed were significantly up- or down-regulated with respect to controls. The two genes related with the JNK pathway (*fos* and *jnk*), which are an evolutionarily conserved signal to regulate cell death and cell proliferation in response to injury were the most severely affected. *runt-1* was also up-regulated more than two-fold. In these analyses we also quantified the expression of *yki*, which is the nuclear effector of the Hippo pathway, involved in tumor progression in mammals and also in planarians^30,31^. Importantly, we show that *yki* is significantly down-regulated in samples exposed to vibration and 4 g (Fig. 4). Maybe the most important result is that four days after the exposure the transcriptional levels of some early genes remained significantly de-regulated in 4 g hypergravity or vibration alone, and even more so in the samples subjected to simultaneous exposure to static and dynamic loads. However, in the latter samples the levels of de-regulation of *fos*, were much lower than the initially very high levels observed in the 0 h samples. It must be also noted that expression levels of *piwi*, which is a marker of stem cells, does not change in a significant manner in any sample. This result suggests that despite the deregulation of the genes required to respond to stimulus, the organism is able to maintain a stable population of stem cells, which also agrees with the observation that planarians can regenerate properly.

Overall, these results indicate that exposure to static and dynamic loads, similar to the ones experienced during a rocket launch produces a high impact in the transcriptional regulation of genes required to properly respond to any injury. Furthermore, the deregulation is reinforced when both elements, hypergravity and vibration, are applied simultaneously, as it occurs during launch. Importantly, the deregulation of the transcripts occurs immediately after the exposure and persists throughout the following days.

## Discussion

Although planarians regenerate an apparent proper head, our results indicate that short exposures to hypergravity and vibration do elicit important transcriptional changes at a genetic level. The deregulation of the early response genes at 0 h indicates that the cells are sensing the ‘stressing’ mechanical stimulus applied and respond by regulating genes that control essential cellular processes as proliferation and cell death. Importantly, although hypergravity or vibration alone already produce transcriptomic alterations, the simultaneous application of both for several minutes produces an even higher alteration of the transcriptional levels of the genes analyzed. The high up-regulation of *fos*, an oncogene involved in the control of cell death in all animal species^32^, indicates the profound effects that this treatment produces in cells. Even more important is the finding that 4 days after returning the animals to normal 1 g conditions the transcriptional levels of some genes analyzed do not reach basal levels. In fact, *egr-1*, and *fos* show a transcriptional alteration that is inverse with respect to the samples at 0 h, which could indicate that a rebound effect is occurring. Hypergravity or vibration alone are also not completely recovered at 4 days. It must be stressed that these alterations are observed in a context of a “whole animal”. Thus, the sustained activation of the early response genes, which main function is to control apoptosis, stem cell proliferation, and differentiation can have an impact in the cell renewal of the organisms affected. The early response genes (*c-myc, c-fos*) expression after launch-related vibrations was also identified by Tjandrawinata and colleagues^33^ where higher levels of both genes were reported 30 min after exposure to a one-axis simulated space shuttle launch (2 min 7.83 *G*_rms_ followed by 6 min at 4.098 *G*_rms_). However, in that cell monolayer in vitro experiment this increase disappeared after 3 h. Using a whole animal like a planarian provides a more realistic situation where we do not see a recovery even after 4 days.

The study by Kumei and coworkers^34^ was one of the very first published experiments that explored the role hypergravity on the expression of early response genes such as *c-fos, c-myc,* and *c-myb*. HeLa cells were exposed for 2–4 days to either 18, 35 of 70 g and only *c-myc* showed an increase in expression. In another study, *c-fos* expression also increased, gradually, in an MC-3T3 bone cell line exposed for 5 min from 50 to as much as 2000 g, while *erg-1* was more expressed at lower g levels^35^. The very first study regarding *c-fos* in real microgravity showed an opposite effect of gravity. In that paper by de Groot and colleagues they reported a strongly decreased epidermal growth factor (EGF)-induced expression of the *c-fos* and *c-jun* proto-oncogenes in a Maser sounding rocket experiment^36^. In the preparation for a spaceflight experiment we identified also the possible anabolic effects of vibration providing the opportunity to elucidate how bone cells (MC-3T3) sense vibration stress^37^. More recent studies explored the effects of parabolic flight-related vibrations on thyroid cancer cells^13^ and the possible anabolic effects in chondrocytes^38^.

Comparing launch vibration simulation with in-flight data Cubano and Lewis concluded that the regulation of heat shock proteins *hsp27* and *hsp70* in Jurkat cells was due to spaceflight^15^. However, based on the current data, it might not be excluded that the effect on *hsp27* could also have been generated by the combined effect of vibration and hypergravity loads during launch. Such a combined test was not performed in that study. In spaceflight experiments, the interest is not generally the effect of the launch, but the effect of microgravity specifically. However, the effect of the launch most likely affects the parameters during the next hours or even days. Thus, understanding what happens during the launch is required to optimize the experimental parameters.

Overall, our results indicate that a relatively short exposure to hypergravity and / or vibration can elicit short-term but also long-lasting cellular responses on a genetic and likely proteomic level. Even if effects of either static g loads or vibration might have no or little effect separately, paradigms such as stochastic residence might transpose sub-threshold responses to a relevant level^39,40^. It would be interesting to explore the nature of the cellular or tissue responses due to vibration. Would the effects be the result of organelle intracellular replacements like the relatively heavier nucleus^37^ or, also depending on the compliance of the experiment volume, a deformation of the whole-cell/tissue body due to inertial gravitational shear^41^. In this respect, it is worth mentioning that *yki* as the effector of the Hippo pathway, which is involved in force transduction of the cellular environment to the nucleus. It has been demonstrated that a direct force on to the cell leads to nuclear translocation of YAP which is the *yki* homolog in vertebrates^42^.

The better experimental design in gravity-related space research is to apply an on-board 1 g centrifuges to control for any spaceflight related effects such as radiation and launch^43^. However, based on current results also the factor time, which is required to fade out launch effects, should also be taken into account by either a delay the in-flight start of an experiment or an implementation of the launch fade out phase by increasing the total active experiment time. This can be combined with the well-established reduction of cellular activity by lowering temperature or reducing medium serum content. During a pilot study in preparation of a sounding rocket study we exposed primary osteoblast to an 11 g launch simulation in the MidiCAR centrifuge at 37 °C. It was shown that cells do sense this profile by phosphorylation of some proteins which could be stopped by ‘launching’ at 8 °C^44^.

This study demonstrated the importance of pre-flight tests especially related to launch effects for life sciences/biological experiments. It might be argued that, especially for institutionally funded basic research experiments, one needs, as part of the qualification process, to demonstrate how a particular experiment responds to launch loads (static, dynamic, and combined) and how this relates to the initiation of the experiment while in microgravity. This is especially true for short-duration microgravity experiments that make use of platforms such as parabolic aircraft^45^, drop towers^46^ or sounding rockets but also the more recent commercial suborbital platforms such as the Blue Origin New Shepard^47^, future Virgin Galactic White Knight^48^, Dream Chaser from Sierra Nevada Company^49^ or the upcoming Space Rider from the European Space Agency^50^. It is even more alarming since our data showed that launch effects are visible directly after lift off until even 4 days post exposure. This indicates that although systems like the Russian Soyuz or the Space-X Dragon are now sometimes coupling to the International Space Station within a day, which has always been favored by the life sciences community, an experiment might still need more time to overcome the launch stress effects before a clear and reliable actual microgravity experiment can be initiated.

We may conclude that in this study, where we exposed whole animals to static g-loads and, for the first time, also used combined launch effects with an actuator located inside a large centrifuge, demonstrated the long-lasting effects of launch loads with a limited number of early response genes. Future experiments should explore the full spectrum of genes and proteins relevant for the research of a particular cell, tissue or animal. Also, we applied a generic launch vibration profile in only one axis, while profiles of the various rockets are different and the location, fixation and/or stowage of the samples during launch are very relevant parameters for the actual vibration profile.

We showed that launch effects and especially the combined static g-loads and dynamic vibration, based on our findings concerning expression of five early response gene’s expressions can be more important, and long lasting than previously expected. Ideally, any space-flight experiment should be exposed to such launch loads and these tests should be made part of the standard flight-related requirements tests an experiment has to go through before an actual study in real microgravity may be performed and produce relevant results.

## Methods

### Planarian culture and exposure to hypergravity and/or vibration

Asexual planarians from a clonal strain of *Schmidtea mediterranea* BCN-10 were maintained at 20 °C in planarian artificial medium (PAM) water, as previously described^51^. Animals were fed with veal liver and starved for at least one week before beginning the experiments. Animals were transported from Barcelona/Spain to ESA-ESTEC Noordwijk/the Netherlands in PAM water using 50 mL falcon tubes.

To study the regeneration process in planarians after exposure to hypergravity and/or vibration animals were amputated (head and tail) one day before the exposure. The day after trunk fragments were loaded into T25 flasks (day 1 of regeneration) (Fig. 1a). Four groups of animals were analyzed: planarians at 1 g without vibration (control), planarians at four times Earth gravity static accelerations without vibration (4 g), planarians at 1 g with dynamic g-loads (vibration), and planarians at 4 g with vibration (Fig. 1B). Three flasks per condition (three replicates), were included with 5 planarians per flask. RNA was extracted from those animals immediately after the exposure (0 h) and 4 days after (4d).

### Simulation of rocket launch

**Fig. 5:**
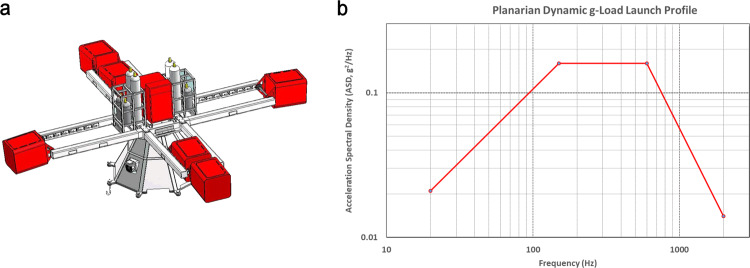
The six red-colored swing-out and one central gondola from the 8-meter diameter Large Diameter Centrifuge (LDC). **a** Both centrifuges are currently located at the technology center (ESTEC) from the European Space Agency ESA) in Noordwijk, the Netherlands. **b** The Acceleration Spectral Density (ASD) of the random vibration test specification profile from the 20 to 2000 Hz range as used for exposing planarians to a simulated launch load. This profile is based on the minimum workmanship levels for random vibration testing^53^. The equipment set-up was divided over two gondolas where the actual actuator was placed in the outer gondola (see for further details Fig. 2).

Planarians were amputated (head and tail) one day before the exposure to launch-related mechanical loads. The day after, trunk fragments were loaded into T25 flasks (day 1 of regeneration). Three T25 flasks were included per condition, and 5 planarians were loaded into each flask. The setup includes the Large Diameter Centrifuge (LDC) (Fig. 5a ref. ^52^), to simulate hypergravity (4 g) and a vibration system, which was bolted to the floor plate of one of the gondolas (Fig. 2). The hardware components of the set-up included the actual shaker model 2075E, a linear power amplifier model 2050E09 both from The Modal Shop (TMS, Cincinnati, OH, USA), a front-end 8-channel data acquisition system (LMS / Siemens SCADAS), a cooling system (Asynchronous Motors Cl. 71\2), and a laptop to control the shaker with the companies dedicated control software. To fix the flasks with planarians on the shaker, an aluminum plate wrapped in a thin rubber sheet was used. The rubber sheet impeded the movement of the flasks along the metal plate. Planarians were subjected to vibration and static hypergravity separately or simultaneously. Four groups of animals were analyzed: planarians at 1 g without vibration (control), planarians at four times Earth’s gravitational force static accelerations without vibration (4 g), planarians at 1 g with dynamic g-loads (vibration), and planarians at 4 g with vibration. The parameters used to simulate launch vibration are shown in Fig. 5b and Table 1.

**Table 1:** Vibration parameters, frequency range and power spectral density, as actually used in the dynamic g exposure of planarians for a period of 8.5 min.

	Maximum acceleration (g)	Maximum velocity (m/s)	Maximum displacement (mm)	Maximum force (*N*)	Frequency (Hz)	Slope (dB/Oct)	Amplitude (g^2^/Hz)
High Vibration	48	0.378	1.42	213	20		0.021
					20–150	3	0.130
					150–600	−6	0.130
					600–2000		
					2000		0.014
Overall *G*_rms_ = **11.28**							

From ref. ^53^

The applied vibration profile was based on the Code of Federal Regulations (CRF) of the Office of the Federal Register National Archives and Record Administration, for launches into space from the Federal Aviation Administration (FAA)^53^. These random vibration tests are usually performed at the payload level of assembly for proto-flight hardware that is subjected to a random vibration test to verify its ability to survive the lift-off environment and also to provide a final workmanship vibration test. In this study we applied that same profile to test the biological responses. The total load experienced by the samples is 11.3 *G*_rms_ in the frequency range between 20 and 2000 Hz (Table 1, Fig. 5b). These vibration levels are also comparable to the acceptance levels profile of the Generalized Random Vibration Test Levels Components (GELV) for payloads of 22.7 kg (50-lb) or less with have an overall *G*_rms_ of 10.0 as are set in NASA standards^54^.

The vibration was applied for only one of the three orthogonal axes and was slightly modified because the shaker could not output the force required by the profile at higher g levels. In the range from 150 to 600 Hz we did lower the power from 0.16 to 0.13 g^2^/Hz. The force was lessened by ~20 Newton. Due to the unorthodox application of an actuator inside a centrifuge, and despite the slight adaptation we made, the high vibration at 4 g ran for 240 s (4 min) at maximum intensity and stopped automatically due to reaching the over-heating settings of the system. We immediately restarted the actuator set-up to run for another 270 s (4.5 min) in order to complete the full launch time simulation of 8.5 minutes.

### Planarian RNA extraction

To perform the transcriptomic analysis through quantitative real-time PCR (qPCR) analysis, total RNA was extracted from the four groups of planarians immediately (0 h) and after four days after exposure (4d). Three replicates each containing a pool of five animals were analyzed per condition. RNA was extracted with Trizol (Invitrogen, Carlsbad, CA, USA), following the manufacturer’s instructions. RNA was quantified with a Nanodrop ND-1000 spectrophotometer (Thermo Scientific, Waltham, MA, USA) and cDNA was synthesized using SuperScript™ III Reverse Transcriptase (Thermo Scientific Waltham, MA, USA) following the manufacturer’s instructions.

### Quantitative real-time PCR

Quantitative PCR’s were performed using *Power* SYBR™ Green PCR Master Mix (Applied Biosystems) on 7500 Real-Time PCR Systems (Applied Biosystems) by denaturation at 95 °C for 10 min, followed by 40 cycles at 95 °C for 15 s and 60 °C for 40 s. Melting curve analyses were performed to verify the amplification specificity. Relative quantification of gene expression was performed according to the ΔΔ-CT method^55^ with at least two technical replicates per sample and at least two biological replicates. The measured C_t_ values were normalized to the ubiquitously expressed control mRNA *smed-ura4*. Student *t*-test was used for differential expression analysis between samples. The set of used primers in qPCR analysis in provided in Table 2.

**Table 2:** Sequence of primers used in the qPCR experiments.

Genes	Primers (5′−3′)	
*runt1*	TCCTATCGGAGACGGACA	GCTTCACCGTTGACGAGT
*egr1*	GTTAGCGTGCCATTTTTGT	AGCTGCATTGATAAGGCTTC
*fos*	GAACGACGCCAATTTCAG	CGCTTCGAGTTGTTTGAGT
*jnk*	TCAACGAATCTCGGTCG	AGTGAGCTCTCTTTCATCAACC
*yorkie*	ATTTGTGTCGACTCCATCC	CCATTAAGACATGTCGACAAG
*piwi*	ATCCTATGGCACCGAATGAG	CCCTTATGCACCTTTCCAAC

### Reporting summary

Further information on experimental design is available in the Nature Research Reporting Summary linked to this paper.

## Supplementary information

Reporting Summary Checklist FLAT

## Data Availability

The data that support the findings of this study are available from the corresponding author upon reasonable request.
